# Paclitaxel-induced germline DNA-damage signatures independent of mismatch repair in *C. elegans*

**DOI:** 10.3389/fphar.2026.1717152

**Published:** 2026-02-18

**Authors:** Yebin Hong, Zifei Liu, Hyun-Min Kim

**Affiliations:** Natural and Applied Sciences, Duke Kunshan University, Kunshan, China

**Keywords:** chemotherapy resistance, DNA repair, germline toxicity, mismatch repair (MMR), multigenerational adaptation, paclitaxel

## Abstract

**Introduction:**

Paclitaxel is a frontline chemotherapeutic agent that stabilizes microtubules, but its broader impact on germline genome stability and long-term tolerance mechanisms remains incompletely understood.

**Methods:**

Using *Caenorhabditis elegans*, we combined phenotypic, cytological, genetic, and transcriptomic approaches, and further validated selected findings in human A498 renal carcinoma cells.

**Results:**

Paclitaxel markedly impaired fertility: hatching dropped approximately 54%, larval arrest increased approximately 4.1-fold, and brood size decreased approximately 31% at 50 μM (all P < 0.01). HIM progeny rose 15-fold, with 52% of nuclei lacking SYP-1 and widespread abnormal chromosome clustering. RAD-51 foci persisted and late-pachytene apoptosis increased 47%, indicating defective DNA damage repair. Transcriptomic analysis revealed upregulation of detoxification and immune pathways and selective downregulation of mismatch repair genes; however, genetic manipulation confirmed that MMR attenuation is a stress signature rather than a causal driver. Multigenerational exposure led to initial sensitization (F5 survival 18.9%) followed by partial recovery (F10 survival 60.1%). Parallel experiments in human kidney cancer cells revealed concentration-dependent apoptosis, reflecting the phenotypes observed in nematodes.

**Conclusion:**

This integrated analysis establishes *C. elegans* as a tractable *in vivo* platform for dissecting both proliferative and meiotic consequences of paclitaxel. By linking spindle disruption, delayed DNA repair, and apoptosis with transcriptional reprogramming, the study provides mechanistic insight into germline vulnerability, reproductive side effects, and potential adaptive responses relevant to chemotherapy.

## Introduction

Chemotherapeutic agents such as paclitaxel (Taxol) are widely used for the treatment of solid tumors owing to their potent ability to stabilize microtubules and block mitotic progression. Beyond their canonical role in disrupting spindle dynamics, paclitaxel and related taxanes have also been implicated in modulating DNA damage responses and DNA repair pathways, thereby influencing both cytotoxicity and drug resistance profiles. Notably, paclitaxel has been reported to interfere with DNA repair processes, including the repair of DNA double-strand breaks, and to synergize with DNA-damaging agents such as cisplatin by slowing lesion repair kinetics (Reed et al., 1995; Dhawan and Swaffar, 1999; Niero et al., 1999; Zhou et al., 2001). Recent studies indicate that paclitaxel impairs DNA repair and promotes DNA damage across cancer models, supporting effects beyond microtubule stabilization (Mohiuddin and Kasahara, 2021; Qu et al., 2023; Morinaga et al., 2024). These observations suggest that the therapeutic effects of paclitaxel may extend beyond microtubule stabilization to the regulation of genome stability, although its exact mechanism remains to be elucidated.

Among the cellular pathways safeguarding genomic integrity, the DNA mismatch repair (MMR) system plays a pivotal role in correcting replication errors and suppressing mutagenesis. Clinical and preclinical studies have revealed a complex and context-dependent relationship between MMR components, such as MSH2 and MLH1, and paclitaxel sensitivity. For instance, MMR deficiency alone does not consistently confer paclitaxel resistance (Fink et al., 1998; Lin and Howell, 1999; Fedier et al., 2001), whereas paradoxically, MSH2 overexpression has been associated with paclitaxel resistance in ovarian cancer models (Zhang et al., 2014). More recent analyses have further suggested that MSH2 upregulation may contribute to paclitaxel resistance signatures, though direct causality remains unresolved (Ge et al., 2025). These findings highlight the possibility that altered MMR activity could modulate the cellular response to paclitaxel-induced stress in unexpected ways.

Despite these intriguing links, the functional interaction between paclitaxel exposure and MMR pathway activity *in vivo* remains poorly understood. This is particularly relevant in the germline, where precise regulation of DNA repair is essential for meiotic chromosome segregation, maintenance of genomic integrity, and reproductive success. The nematode *Caenorhabditis elegans (C. elegans)* offers a powerful model to address these questions, as its germline architecture enables spatiotemporal dissection of meiotic progression, DNA damage responses, and apoptosis, while genetic tools permit targeted manipulation of MMR genes.

Using *C. elegans* as a model, we systematically investigated paclitaxel-induced meiotic defects, including impaired chromosome synapsis, delayed double-strand break repair, apoptosis, and transcriptional reprogramming - including downregulation of DNA mismatch-repair genes that, contrary to expectation, do not drive embryonic lethality but rather constitute a stress signature.

## Materials and methods

### Strains and alleles

All *C. elegans* strains were maintained at 20 °C under standard laboratory conditions, as previously described (Brenner, 1974). The N2 Bristol strain was used as the wild-type reference.

### Phenotypic assays

Age-matched young-adult hermaphrodites (∼24 h post-L4) were used for phenotypic assays. Brood size was defined as the total number of eggs laid by a single animal; embryonic lethality as the percentage of those eggs that failed to hatch within 24 h of being laid; larval arrest/lethality as the percentage of hatched larvae that did not grow/survive to adulthood; and the Him phenotype as the percentage of male adults among the total adult progeny as described previously (Kim and Colaiacovo, 2014; Kim and Colaiacovo, 2015). For each genotype and condition, at least 20 hermaphrodites were scored in every generation. Statistical comparisons between groups were performed with the two-tailed Mann–Whitney test at a 95% confidence interval.

### Paclitaxel treatment and concentration rationale

To assess the effects of paclitaxel in *C. elegans*, young adult worms were exposed to paclitaxel added to NGM plates at concentrations of 0, 25 and 50 µM for 20–24 h. This concentration range was selected based on human pharmacokinetic data and predicted drug accumulation in worms. In clinical settings, the most commonly used dose of cremophor-diluted paclitaxel (175 mg/m^2^ over a 3-h infusion) produces a median maximum plasma concentration (Cmax) of approximately 5.1 µM (interquartile range: 4.5–5.7 µM) (Stage et al., 2018). Because *C. elegans* absorbs only a fraction of compounds from the environment—fewer than 10% of small molecules accumulate to more than 50% of their plate concentration (Burns et al., 2010)—plate concentrations of 25–50 µM were used to achieve estimated intracellular levels of 2–5 μM, approximating the human Cmax. This approach ensures that the worms are exposed to pharmacologically relevant concentrations, while accounting for the limited uptake efficiency.

### RNA sequencing analysis

Synchronized *C. elegans* populations at 1 day post-L4 stage were collected, washed three times with M9 buffer, and pelleted. Total RNA was extracted using TRIzol reagent and treated with DNase I to remove genomic DNA contamination. RNA concentration and purity were assessed using NanoDrop and Qubit 2.0, and integrity was verified with the Agilent 2,100 Bioanalyzer. Only high-quality RNA samples were used for library preparation.

mRNA was isolated using Oligo (dT)-attached magnetic beads and fragmented, followed by first- and second-strand cDNA synthesis. cDNA fragments underwent end repair, adapter ligation, size selection (300–400 bp), and PCR amplification to construct sequencing libraries. Library quality and concentration were evaluated using Qubit, Agilent 2,100, and qPCR. Libraries were pooled and sequenced on the Illumina platform by BMKGENE (Beijing, China).

Raw reads in FASTQ format were filtered to remove adapter sequences and low-quality bases, producing high-quality clean data (≥6.93 Gb per sample, ≥93.36% bases Q30). Clean reads were mapped to the *C. elegans* reference genome (WS279), with mapping ratios ranging from 97.80% to 98.34%. Gene expression levels were quantified and normalized to account for library size and sequencing depth. Differentially expressed genes (DEGs) were identified using the criteria of Fold Change ≥2 and P-value <0.05. All experiments included at least three biological replicates per condition. Library quality, sequencing depth, and base quality were monitored by BMKGENE to ensure data reliability and reproducibility.

### Paclitaxel sensitivity assay

For paclitaxel sensitivity assays, L4 or young adult animals were exposed to 0, 25, or 50 µM paclitaxel on NGM agar plates seeded with *E. coli* OP50 and kept in the dark for ∼20 h. Following treatment, animals were washed with M9 buffer, then transferred to fresh NGM plates to lay eggs for 3–6 h. Embryonic hatching was assessed ∼24 h later. Each treatment condition was replicated at least twice in independent experiments.

### Multigenerational paclitaxel exposure and recovery assay

To test for drug adaptation, we exposed animals to paclitaxel across ten successive generations (F1–F10). For each generation, 50–100 young-adult hermaphrodites were transferred to NGM plates containing 0, 25 or 50 µM paclitaxel and allowed to lay embryos for 24 h at 20 °C on fresh NGM (no paclitaxel) and cultured to adulthood at 20 °C. Progeny that developed under these drug-free conditions were then used as parents for the next cycle; the same 20–24 h paclitaxel pulse (25 or 50 µM) was repeated for every generation. Experiment was performed at least twice independently.

### Immunofluorescence staining

Immunofluorescence staining of whole-mount gonads was performed as described in (Colaiacovo et al., 2003; Kim and Colaiacovo, 2014). Primary antibodies used included rabbit anti-RAD-51 (1:2000; SDIX), rabbit anti-SYP-1 (1:250 (MacQueen et al., 2002)), and secondary antibodies were Cy3 anti-rabbit (1:300, Jackson Immunochemicals). Fluorescence images were captured at 0.2 μm intervals using an Eclipse Ti2-E inverted microscope equipped with a DSQi2 camera (Nikon) or CSU-X1. Images were obtained with a ×100 objective and subjected to deconvolution using NIS Elements software (Nikon) or VisiView. Partial projections of half-nuclei are shown.

### Generation of the *msh-2* over-expression strain

A genomic fragment spanning the entire *msh-2* coding region was amplified from *C. elegans* N2 genomic DNA (Q5 High-Fidelity DNA polymerase, NEB) and cloned into pPD95.77 via Gibson assembly so that it is flanked by the *pie-1* promoter and the *msh-2* 3′UTR. The resulting plasmid was co-injected with the fluorescent marker pCFJ90 into the gonad syncytium of young adult hermaphrodites. F1 worms were UV-irradiated (300 J m^-2^, Stratalinker 2,400) to induce integration, and integrants were out-crossed to wild-type males four times. Stable transgenic lines were maintained for >6 generations before use. Over-expression of *msh-2* was verified by qPCR (Figure 5A) using *tba-1* as the reference gene.

### Quantitation of germline apoptosis

Germline apoptosis was assessed by acridine orange staining in age-matched (∼20 h post-L4) animals, following the method described in (Kelly et al., 2000; Kim and Colaiacovo, 2014). A Nikon Ti2-E fluorescence microscope was used to score between 20 and 30 gonads per treatment. Statistical significance was determined using the two-tailed Mann–Whitney test, with a 95% confidence interval.

### Quantitative real-time PCR

cDNA was synthesized from RNA extracted from young hermaphrodite worms using the ABscript II First Strand synthesis kit (ABclonal, RK20400) as described previously (Meng et al., 2023). Real-time PCR was performed using ABclonal 2X SYBR Green Fast Mix (RK21200) in a LineGene 4,800 (BIOER, FQD48A) or Biorad CFX real-time system. The initial denaturation step was carried out at 95 °C for 2 min, followed by 40 cycles of 95 °C for 15 s, 60 °C for 20 s, and elongation. A melting curve analysis (60 °C–95 °C) was conducted to verify the specificity of the PCR products. Tubulin encoding gene *tba-1* was used as a reference gene based on microarray data for *C. elegans*. Each PCR experiment was repeated at least twice.

### RNAi

Feeding RNAi experiments were performed at 20 °C as described (Zhang et al., 2021) using the Ahringer RNAi library clone. HT115 bacteria carrying the empty pL4440 vector were used as control RNAi. cDNA was produced from single-worm RNA extracts using the ABscript II First synthesis system (ABclonal RK20400). RNAi effectiveness was examined by assaying the expression of the transcript being depleted in four individual animals subjected to RNAi by feeding. Expression of either the *tba-1* transcripts was used as a control.

## Results

### Paclitaxel’s impact on meiotic segregation and fertility

**FIGURE 1:**
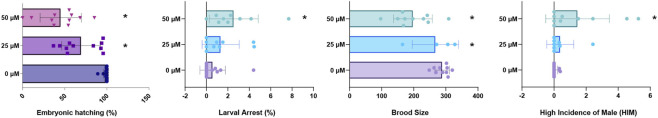
Exposure to Paclitaxel alters embryonic hatching, Larval arrest, HIM phenotype, and fertility. The figure depicts the phenotypic outcomes of *C. elegans* exposed to various doses of paclitaxel. Assessed parameters include embryonic hatching rate, larval arrest/lethality, brood size, and the incidence of the HIM phenotype (% males). Error bars represent the standard error of the mean (SEM). Statistical significance relative to the untreated control was assessed using a two-tailed Mann–Whitney U test. Asterisks denote statistically significant differences (P < 0.05). For each assay, n = 24 independent animals per condition.

To evaluate the potential toxicity of paclitaxel, age-matched adult hermaphrodites were exposed to paclitaxel for approximately 24 h and embryonic lethality was monitored as in (Kim and Colaiacovo, 2015). Exposure to paclitaxel led to a significant 54% reduction in hatching (Figure 1, 98.8% vs. 45% in 0 and 50 μM, P < 0.0001 by Mann-Whitney test) as well as ∼30% reduction at 25 μM (98.8% vs. 69.5% in 0 and 25 μM, P < 0.0001 by Mann-Whitney test).

Moreover, worms exhibited a 4.1-fold increase in larval arrest/lethality following paclitaxel exposure (0.6 vs. 2.5 in 0 and 50 μM, P = 0.0047). ∼2.5% of L1-L3 stage larvae did not survive until adulthood after 50 μM of paclitaxel, suggesting that paclitaxel disrupts larval development. Paclitaxel-treated worms exhibited a 31% reduction in brood size compared to the untreated group, indicating sterility (288 vs. 197 in 0 and 50 μM, P = 0.0022).

In *C. elegans,* aberrant sex chromosome segregation led to high incidence of male (HIM) which is an indicator of disturbed meiotic development. The exposure to Paclitaxel resulted in a 15-fold increase in the frequency of the HIM phenotype, suggesting a disruption in meiotic chromosome segregation (0.1 vs. 1.5 in 0 and 50 μM, P = 0.0221).

Collectively, these findings underscore the significant impact of Paclitaxel on both meiotic chromosome segregation and mitosis, leading to embryonic and larval arrest/lethality, reduced fertility, and a higher incidence of male progeny. To further characterize meiotic progression at the cellular level, we next examined germline morphology.

### Paclitaxel-mediated germline abnormalities extend beyond proliferative defects to meiotic dysfunction

In *C. elegans*, nuclei are arranged in a spatial and temporal gradient along the germline, facilitating the identification of abnormal chromosomal organization during specific meiotic stages (Lui and Colaiacovo, 2013). To investigate whether the increased sex chromosome nondisjunction and reduced fertility is due to defective meiotic defects, we conducted a comparative morphological analysis following exposure to paclitaxel.

**FIGURE 2:**
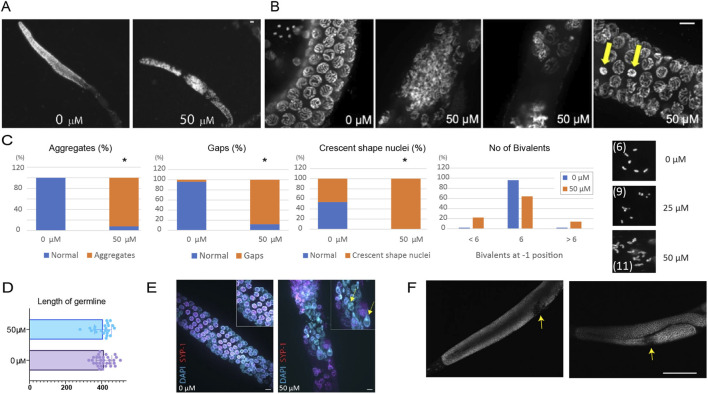
Paclitaxel induces meiotic abnormalities in the germline. **(A)** Representative DAPI-stained gonads from untreated and paclitaxel-treated worms. **(B)** Distinct phenotypes were observed upon paclitaxel exposure, including nuclear aggregates, increased inter-nuclear gaps, and crescent-shaped nuclei (arrows). Please see Supplementary Figure S1 for the time-course observations. **(C)** Quantification of meiotic phenotypes described in **(B)**. Bar graphs show the percentage of nuclei per gonad exhibiting aggregates, gaps, crescent-shaped nuclei and number of bivalents. Statistical significance was determined using a two-tailed Mann-Whitney test (*p < 0.05) comparing paclitaxel-treated worms (∼24 h 50 μM) to untreated controls (0 μM). Paclitaxel treatment significantly increased aggregates, gaps, and crescent-shaped nuclei. Representative images of misaligned/fragmented versus intact bivalents are shown on the right. **(D)** Measurement of germline length shows no significant difference between treated and untreated worms. **(E)** SYP-1, a component of the synaptonemal complex, co-localizes with DAPI-stained nuclei in control worms but is absent in 52% of nuclei in paclitaxel-treated gonads (arrows). Scale bars = 10 μm. **(F)** Two representative DAPI-stained images of adult gonad arms ∼24 h after 50 µM paclitaxel exposure. In hyperproliferative *glp-1(ar202)* mutants, paclitaxel induced visible gaps along the gonad arm, whereas untreated gonads remained intact. Scale bar = 50 µm. Statistical differences were determined using a two-tailed Mann–Whitney test; error bars indicate SEM. For all quantified assays, n ≥ 24 animals per condition.

While untreated control worms displayed well-organized nuclei (Figures 2A,B, 0 μM), worms exposed to paclitaxel exhibited distinct characteristics, including the presence of aggregates consisting of two or more nuclei, increased inter-nuclear spacing, and the appearance of crescent-shaped nuclei which are typically observed during the leptotene or zygotene stage but were now also present during the pachytene stage (Figures 2B,C; Supplementary Figure S1).

We found that all three phenotypes were statistically significant compared with the untreated control (percentage of nuclei with aggregates: 0% vs. 92% at 0 and 50 μM, P < 0.0001; percentage of nuclei showing a gap distance >10 μm: 4% vs. 88% at 0 and 50 μM, P < 0.0001; percentage of crescent-shaped nuclei: 46% vs. 100% at 0 and 50 μM, P < 0.0001). In addition, treatment with paclitaxel resulted in a higher frequency of misaligned or fragmented bivalents and a lower frequency of intact bivalents.

In contrast, the overall germline length remained unchanged (Figures 2D, 402.6 μm vs. 387.4 μm at 0 and 50 μM, P = 0.8312), indicating that the defects are not attributable to alterations in tissue size or growth.

To further validate defective meiotic progression, we further analyzed the localization of the synaptonemal complex during meiotic development. SYP-1 is a central component of the synaptonemal complex, which forms between homologous chromosomes during meiosis. In the control group, we observed co-localization of DAPI-stained nuclei and SYP-1 (Figure 2E, 100%, 1,000 out of 1,000 nuclei). However, in paclitaxel-treated nuclei, there was often a lack of SYP-1 localization (52%, 521 out of 1,000), suggesting defective homologous chromosome pairing and meiotic development.

Collectively, these results support the conclusion that paclitaxel-induced microtubule stabilization impairs meiotic spindle dynamics, leading to defective chromosome alignment, synapsis failure, and ultimately germline aneuploidy.

We next asked whether paclitaxel exposure elicits growth defects in genetic contexts that mimic hyperproliferative, cancer-like germlines. In *glp-1(ar202*) (gain-of-function) mutants, paclitaxel disrupted germline progression, producing visible gaps along the gonad arm, whereas control animals showed no comparable defects (Supplementary Table S1; Figure 2F).

Quantification revealed disruptions in 60% of paclitaxel-treated gonads versus 5% (1/20) in untreated controls. In the hyperproliferative *glp-1(ar202)* background—where every distal nucleus is maintained in a stem-cell-like, mitotically competent state (Hubbard and Schedl 2019)—these disruptions occurred in a population containing both actively dividing and proliferation-prone germ-cell nuclei. This suggests that paclitaxel’s toxicity affects more than just nuclei undergoing active proliferation.

Together, these results reveal that paclitaxel disrupts germline integrity not only by impairing proliferative nuclei but also by inducing profound meiotic defects. Beyond its known interference with mitotic progression, our data are consistent with paclitaxel stabilizing microtubules in a manner that hinders microtubule-based chromosome alignment and synapsis. This dual impact on both proliferative and meiotic compartments highlights paclitaxel as a potent disruptor of germline genome stability and underscores its broader implications for fertility and aneuploidy.

### Paclitaxel disrupts DSB repair and promotes conserved apoptotic responses in *C. elegans* and human cells

**FIGURE 3:**
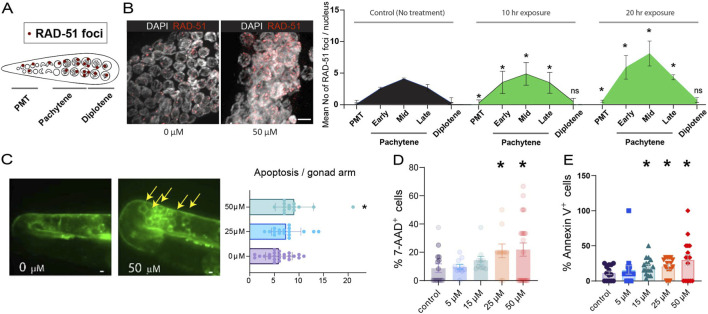
Paclitaxel induces an elevated level of RAD-51 foci formation and germline apoptosis, and reduces viability of human cancer cells. **(A)** Diagram of germline zones scored for RAD-51 foci. **(B)** Representative mid-pachytene images taken 20–24 h after paclitaxel treatment (50 μM). Right, quantification of RAD-51 foci across germline zones after ∼10 and ∼20 h of exposure. Paclitaxel-treated worms exhibited significantly higher RAD-51 foci in mitotic and meiotic nuclei compared to controls, except at diplotene where levels converged. Mean RAD-51 foci per nucleus are presented in different zones. Asterisks denote statistical significance (*p < 0.05; ns = not significant) determined by a two-tailed Mann-Whitney test with a 95% confidence interval, comparing 50 μM paclitaxel to control at each germline zone. n ≥ 20 gonads per condition from three independent biological replicates. **(C)** Apoptosis quantification using CED-1::GFP reporter. Paclitaxel increased apoptotic nuclei (arrow) by ∼47% compared with controls at 20–24 h post-treatment. Scale bars = 10 μm. Data represent mean ± SEM; n ≥ 5 gonads per condition from three independent biological replicates. Statistical significance determined by Mann–Whitney test. **(D)** Dose-dependent cytotoxicity of paclitaxel in A498 human renal carcinoma cells. Cells were treated with 0–50 µM paclitaxel for 20 h, and membrane-compromised populations were quantified by 7-AAD^+^ exclusion. A significant increase in non-viable cells was detected at concentrations ≥25 µM. Data represent mean ± SEM from ≥3 independent experiments. Raw images are provided in Supplementary Figure S2. **(E)** Apoptosis induction in A498 cells 20–24 h after paclitaxel treatment, assessed by annexin V^+^–FITC staining. Paclitaxel significantly increased apoptotic fractions at 15–50 µM. Data represent mean ± SEM from ≥3 independent experiments. Statistical significance was determined by two-tailed Mann–Whitney test (*P* < 0.05). Raw images are provided in Supplementary Figure S2.

Accumulation of unrepaired double-strand breaks (DSBs) can trigger a DNA damage checkpoint, resulting in increased levels of apoptosis during the late pachytene stage in the *C. elegans* germline (Gartner et al., 2008; Kim and Colaiacovo, 2014). Given the various observed defective meiotic progressions in the germline, we conducted tests to determine whether paclitaxel hampers the double-strand breaks repair process. We quantified the levels of RAD-51 foci, which mark sites undergoing DSB repair, in both mitotic (premeiotic tip/PMT) and meiotic nuclei (Figure 3A). These levels were compared between the control and 50 μM paclitaxel-treated worms.

After 10 and 20 h of paclitaxel exposure, significantly elevated levels of RAD-51 foci were observed throughout the germline compared to untreated controls (Figure 3B). At the premeiotic tip, RAD-51 foci increased from 0.30 foci per nucleus at 10 h (P = 0.0116) to 0.53 foci per nucleus at 20 h (P = 0.0158), indicating an early onset of DNA damage response. This elevation persisted across meiotic stages, with significantly higher RAD-51 foci observed at early, mid, and late pachytene in both exposure conditions. Specifically, paclitaxel-treated worms exhibited averages of 3.46, 4.78, and 3.35 foci per nucleus at 10 h (P = 0.0079, P = 0.0173, and P = 0.0099), and 5.89, 8.10, and 4.34 foci per nucleus at 20 h (P = 0.007, P = 0.004, and P = 0.004), respectively. By the diplotene stage, RAD-51 levels converged with control levels in both treatments (0.33 and 0.73 foci/nucleus for 10 h and 20 h, P = n. s.), suggesting that unrepaired DSBs are eventually resolved or eliminated through apoptosis. The comparable patterns observed at 10 h and 20 h indicate that paclitaxel rapidly triggers defects in DSB repair progression, with persistent RAD-51 accumulation reflecting a time-dependent delay in meiotic repair dynamics rather than an acute, transient response.

We reasoned that defective meiotic progression may trigger elevated germline apoptosis. To test this, we quantified apoptotic nuclei using the CED-1::GFP reporter, which marks cell corpses through the engulfment receptor CED-1 (Figure 3C). Paclitaxel treatment significantly increased apoptosis, from an average of 5.9–7.4 nuclei (in 0 and 25 μM). At 50 μM, apoptosis was further elevated by ∼47% compared to untreated controls (5.9 vs. 9.0 nuclei, P = 0.0070), supporting the notion that paclitaxel-induced meiotic defects promote germline apoptosis.

Taken together, these data support the conclusion that paclitaxel disrupts the normal progression of DSB repair in both mitotic and meiotic germline nuclei, ultimately promoting elevated germline apoptosis in *C. elegans*.

Given the clinical relevance of paclitaxel as an anticancer agent, we next asked whether similar cytotoxic and pro-apoptotic effects could also be observed in human cancer cells. To address this, we next examined A498 renal carcinoma cells under the same concentration range. A498 cells exposed to paclitaxel (0–50 μM, 20 h) showed a dose-dependent increase in both viability loss (7-AAD^+^) and apoptosis (annexin V^+^). Membrane-compromised cell fractions remained unchanged at 5 and 15 μM, but rose significantly at 25 µM (Figure 3D, 20.0%, P = 0.0324) and 50 µM (23.6%, P = 0.0465). Similarly, apoptosis was significantly elevated at 15 µM (Figure 3E, 16.9%, P = 0.0348), 25 µM (20.8%, P = 0.0310), and 50 µM (28.8%, P = 0.0325) compared to control.

Thus, paclitaxel was associated with concentration-dependent apoptosis in both *C. elegans* germline and human carcinoma cells, with significant effects emerging at ≥25 µM. While the underlying mechanisms may differ between systems, the broadly similar dose–response trends support the translational relevance of our experimental approach.

### Paclitaxel rewires the *C. elegans* germline transcriptome: mismatch repair down-modulation

**FIGURE 4:**
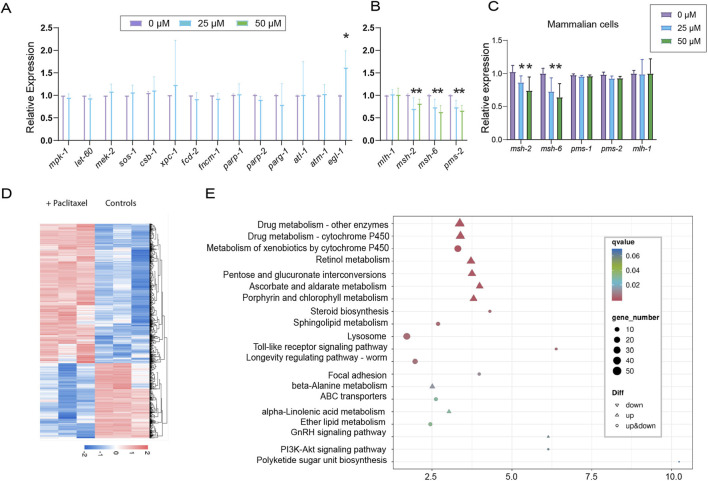
Paclitaxel Rewires the C. elegans Germline Transcriptome: MMR Down-Modulation, and Xenobiotic-Metabolism/TLR-Pathway Activation. **(A)** Expression analysis of genes representing multiple signaling and DNA damage–response pathways, including MAPK signaling (*mpk-1, let-60, mek-2, sos-1*), nucleotide excision repair (*csb-1, xpc-1*), Fanconi anemia (*fcd-2, fncm-1*), poly (ADP-ribose) polymerases (*parp-1, parp-2*), poly (ADP-ribose) glycohydrolase (*parg-1*), ATR (*atl-1*), ATM (*atm-1*), and apoptosis (*egl-1*), in wild-type worms treated with 0, 25, or 50 µM paclitaxel. Asterisks denote statistical significance, indicating differences between the untreated and treated groups as determined by the Mann-Whitney test. **(B)** Quantitation of the expression levels of key *C. elegans* genes involved in the mismatch repair pathway. **(C)** Quantitation of the expression levels of key human genes involved in the mismatch repair pathway. **(D)** Hierarchical clustering of differentially expressed genes in control and 25 µM paclitaxel-treated groups. Red and blue colors represent upregulation and downregulation, respectively, based on the fold change in gene expression. Gene expression levels (measured in FPKM) were normalized using a log10 transformation and are depicted in various colors according to the provided scale bar. **(E)** Pathway enrichment analysis reveals upregulation of xenobiotic metabolism and TLR-mediated immune responses. Data are presented as mean ± SD from ≥2 independent experiments for panels **(A–C)** and from *n* = 3 independent biological samples for panels **(D)** and **(E)**.

Given the observed meiotic and DNA repair defects, we quantified gene expression changes by qPCR. Genes involved in MAPK signaling, nucleotide excision repair, PARP-mediated repair showed no significant alteration (Figure 4A, P = 0.801 for *mpk-1*, P = 0.707 for *csb-1*, P = 0.879 for *parp-1*). Similarly, Fanconi anemia genes showed no significant alteration (P = 0.721 for *fcd-2*, P = 0.843 for *fncm-1*). In contrast, the pro-apoptotic regulator *egl-1* was significantly upregulated (1.62-fold, P = 0.044), indicating activation of apoptosis.

Interestingly, components of the mismatch repair (MMR) pathway were selectively downregulated following paclitaxel treatment in a dose-dependent manner (Figure 4B). Specifically, *msh-2* expression decreased by 30% at 25 μM (P = 0.0027) and 19% at 50 μM (P = 0.0021), while *msh-6* was reduced by 26% and 37% at 25 and 50 μM, respectively (P = 0.0003 and P = 0.0006). Similarly, *pms-2* expression declined by 26% at 25 μM and 33% at 50 μM (P = 0.0011 and P = 0.0017). In contrast, *mlh-1* remained largely unaffected, showing only 2.5% and 1.5% reductions at 25 and 50 μM, respectively (P = 0.1714 and P = 0.3143). These results suggest that expression of MMR components is reduced upon paclitaxel treatment.

To test conservation in human cells, MMR gene expression was measured following paclitaxel treatment. Consistent with *C. elegans*, *msh-2* and *msh-6* were downregulated at 25 and 50 μM. At 50 μM, *msh-2* decreased by 27% (Figure 4C, P = 0.0466) and *msh-6* by 36% (P = 0.0160). In contrast, *pms-1, pms-2,* and *mlh-1* were not significantly altered (P = 0.1706, P = 0.0556, P = 0.9166, respectively).

Altogether, these data demonstrate that paclitaxel induces pro-apoptotic signaling via *egl-1* activation while selectively impairing MMR pathways in both *C. elegans* and human cells, revealing a conserved mechanism of targeted DNA repair disruption.

### Transcriptome-wide gene expression changes: paclitaxel-induced activation of xenobiotic metabolism and TLR pathways

Expanding these observations, RNA-seq analysis of worms treated with 0, 50 μM paclitaxel revealed broad transcriptional rewiring. Hierarchical clustering of control versus 50 µM paclitaxel-treated worms highlighted distinct transcriptional patterns (Figure 4D).

Pathway analysis revealed coordinated upregulation of xenobiotic metabolism and innate immune signaling. Genes associated with drug metabolism, including cytochrome P450 enzymes and other xenobiotic-metabolizing enzymes, were significantly upregulated (Figure 4E). Additional metabolic pathways, including retinol, ascorbate and aldarate, pentose and glucuronate interconversions, and porphyrin and chlorophyll metabolism, were also enriched, reflecting cellular detoxification responses. Concurrently, genes in the toll-like receptor (TLR) signaling pathway were differentially regulated, indicating activation of innate immune responses to mitigate paclitaxel-induced stress.

Together, these data reveal that paclitaxel rewires the *C. elegans* germline transcriptome through two concurrent signatures: upregulation of pro-apoptotic signaling (*egl-1*) and transcriptional down-modulation of mismatch-repair genes (*msh-2, msh-6, pms-2*); we further tested whether the latter change is causal or merely correlative.

### Paclitaxel-induced embryonic lethality occurs independently of mismatch repair gene expression

Following paclitaxel treatment, *msh-2* expression was reduced, and the treated worms exhibited high embryonic lethality. This raised the question of whether reduced level of *msh-2* contributed to phenotypes exhibited in the paclitaxel exposed worms. To determine whether reduced *msh-2* expression contributes to paclitaxel sensitivity, we assessed the correlation between *msh-2* levels and the observed phenotypes. Specifically, we investigated whether restoring *msh-2* expression could mitigate paclitaxel-induced effects.

**FIGURE 5:**
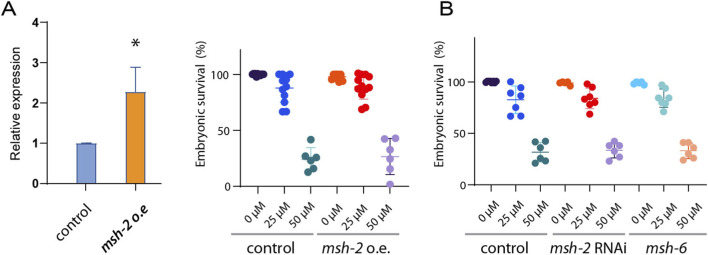
Manipulation of mismatch repair components does not alter paclitaxel-induced embryonic lethality in *C. elegans*. **(A)** qPCR validation of *msh-2* overexpression. Transcript levels of *msh-2* in the Ppie-1-*msh-2* transgenic strain (*o.e*) are shown relative to the control, normalized to the mean of reference gene (*tba-1*). Right, Overexpression of *msh-2* (Ppie-1-*msh-2* gDNA-*msh-2* 3′UTR) did not rescue paclitaxel-induced embryonic lethality. Embryonic survival was comparable between wild type and *msh-2* overexpressing worms at both 25 μM and 50 μM paclitaxel. **(B)** Knockdown of *msh-2* by RNAi similarly did not exacerbate paclitaxel sensitivity. Embryonic survival was 82.7% vs. 84.1% at 25 μM (P = 0.4508) and 31.7% vs. 33.5% at 50 μM paclitaxel (P = 0.3740) for wild type and *msh-2* RNAi worms, respectively. Right, Deletion of *msh-6* (*pk2504*) also had no significant effect on paclitaxel sensitivity. Data are presented as mean ± SEM from ≥2 independent biological samples per condition. Statistical significance was determined using a two-tailed Mann–Whitney test.

To test whether paclitaxel-induced downregulation of mismatch repair (MMR) genes directly contribute to developmental defects, we manipulated *msh-2* expression in *C. elegans*. We generated a germline-specific overexpression strain (Ppie-1-*msh-2* gDNA-*msh-2* 3′UTR) and exposed these worms to paclitaxel. qPCR analysis confirmed that *msh-2* transcript levels in the transgenic worms were 2.3-fold higher than in the control (P = 0.0012, Figure 5A). Overexpression of *msh-2* did not alter embryonic lethality compared to wild-type controls. At 25 μM paclitaxel, embryonic survival was 87.9% in wild-type versus 88.9% in *msh-2* overexpressing worms (Figure 5A, P > 0.45), and at 50 μM survival was 24.3% versus 26.6% (P > 0.35), indicating that restoring *msh-2* expression does not rescue paclitaxel-induced phenotypes.

Conversely, reduction of *msh-2* expression by RNAi also failed to modify paclitaxel sensitivity. While both groups showed nearly 100% embryonic survival under untreated conditions, embryonic survival at 25 μM was 82.7% in wild-type and 84.1% in *msh-2* (RNAi) worms (Figure 5B, P > 0.45), and at 50 μM survival was 31.7% versus 33.5%, respectively (P > 0.35).

Consistent with these findings, deletion of another downregulated MMR component, *msh-6 (pk2504*), also had no significant impact on embryonic survival. At 25 μM paclitaxel, embryonic survival was 82.7% in wild-type and 84.2% in *msh-6* mutants (P = 0.5), and at 50 μM survival was 31.7% versus 33.0% (P = 0.4680).

Collectively, these findings suggest that the reduction in expression of key mismatch repair components, including *msh-2* and *msh-6*, is unlikely to be the primary driver of paclitaxel-induced embryonic lethality. This conclusion is supported by the observation that neither overexpression nor loss of these genes significantly altered the developmental defects. Instead, our data are more consistent with the idea that MMR downregulation reflects a stress-associated transcriptional signature secondary to paclitaxel exposure. Nonetheless, we cannot exclude the possibility that altered MMR expression contributes in more subtle or context-dependent ways.

### Progressive sensitivity followed by partial recovery under continued paclitaxel

**FIGURE 6:**
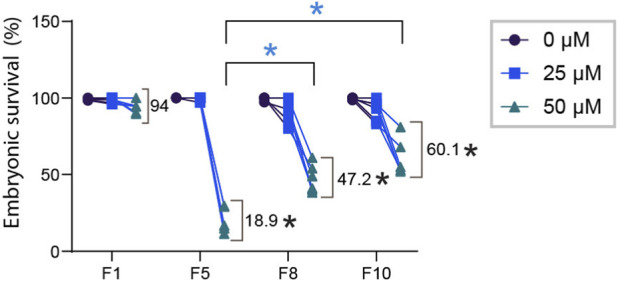
Acquisition of paclitaxel tolerance across generations. Embryonic survival was measured in *C. elegans* populations continuously exposed to paclitaxel from F1 to F10. While the F1 generation maintained 94% survival, survival was dramatically reduced to 18.9% by F5, indicating high sensitivity. With continued exposure, embryonic survival significantly recovered to 47.2% in F8 and further increased to 60.1% in F10. Statistical analyses were performed using the Mann–Whitney test. Comparisons with F1 are denoted by black asterisks (p < 0.0001), and recoveries at later generations are indicated by blue asterisks (p < 0.0001). Data are presented as mean embryonic survival percentages ±SEM from 2 independent biological replicates.

Given that MMR downregulation is not responsible for paclitaxel sensitivity, we next asked whether worms can adapt to paclitaxel over multiple generations. We further investigated how paclitaxel tolerance is acquired over successive generations. While the F1 generation maintained 94% embryonic survival, continuous exposure led to a dramatic reduction to 18.9% survival by F5, indicating pronounced sensitivity (Figure 6, Mann–Whitney test, p < 0.0001, comparisons with F1 are denoted by black asterisks). However, with continued exposure, embryonic survival partially recovered to 47.2% in F8 (p < 0.0001 vs. F1), and further increased to 60.1% in F10 (p < 0.0001 vs. F1, These recoveries are indicated by blue asterisks) suggesting that prolonged paclitaxel exposure promotes partial tolerance through adaptive or selective processes, rather than a full restoration of resistance.

## Discussion

This study systematically investigated the impact of paclitaxel on genome stability using the *C. elegans* germline as a model system. In summary, we found that paclitaxel (1) reduces fertility and increases levels of embryonic lethality, larval arrest/lethality, and HIM (high incidence of males) frequency; (2) induces meiosis-specific defects, including SYP-1 depletion, abnormal bivalent alignment, and increased nuclear clustering and gaps; (3) causes defects in DSB repair, indicated by persistent RAD-51 accumulation; (4) activates apoptotic signaling, while selectively downregulating the transcription of mismatch repair (MMR) components (*msh-2*, *msh-6*, *pms-2*); (5) however, neither *msh-2* overexpression, *msh-2* RNAi knockdown, nor *msh-6* mutation altered embryonic susceptibility, indicating that reduced MMR expression is more likely a downstream consequence of paclitaxel exposure rather than a direct causal driver of toxicity. (6) Under repeated multigenerational exposure, we observed an initial sensitization followed by partial recovery. While this trend suggests possible adaptive responses, we cannot exclude alternative explanations such as selective survival of resistant individuals or experimental variability. Below, we interpret these results in the context of prior literature, propose potential mechanistic connections, and discuss their implications with appropriate caution. We note that paclitaxel has broad pleiotropic effects on both mitotic and meiotic cells, complicating the assignment of causal pathways for the observed phenotypes.

### Dual targeting of paclitaxel—disruption of both mitosis and meiosis

Our data also show that paclitaxel disrupts not only the canonical “microtubule stabilization → mitotic spindle defects” pathway but also directly perturbs meiotic processes. These observations likely reflect pleiotropic defects arising from both mitotic spindle disruption and downstream meiotic errors, making it difficult to assign a single causal pathway for the observed abnormalities. Loss of SYP-1, crescent-shaped nuclear morphology, and fragmented bivalents during pachytene indicate compromised homologous synapsis and alignment, while increased HIM frequency reflects nondisjunction of sex chromosomes during meiosis. These observations are consistent with studies in mammalian cells and animal models showing that paclitaxel perturbs spindle dynamics and induces cell death, and align with clinical and preclinical evidence of enhanced DNA damage and synergistic toxicity when combined with radiation or cisplatin (Reed et al., 1995; Niero, Emiliani et al., 1999).

### Mechanistic linkage: spindle disruption → synapsis failure → DSB repair delay → checkpoint/apoptosis

Programmed DSBs during meiosis must be efficiently repaired in concert with synaptonemal complex formation. The significant increase in RAD-51 foci across meiotic zones, particularly in pachytene, together with elevated apoptosis in late pachytene, supports a model in which paclitaxel-induced spindle and chromosomal alignment defects delay DSB repair, activate DNA damage checkpoints, and induce apoptosis via *egl-1* upregulation (Zhou et al., 2001). Thus, microtubule stabilization not only blocks physical segregation but also interferes with meiosis-specific repair and synchronization processes. These defects likely arise from combined mitotic and meiotic disruptions, underscoring the pleiotropic effects of paclitaxel in the germline.

Paclitaxel-induced microtubule stabilization can also impair the nuclear trafficking of DNA repair proteins. Disruption of dynein-mediated import causes cytoplasmic sequestration of key homologous recombination factors such as ATM, ATR, and the MRN complex (Poruchynsky et al., 2015; Markowitz et al., 2017). This defect may specifically delay post–strand invasion steps of DSB repair, as RAD-51 filament disassembly requires nuclear HELQ-1 helicase and RFS-1 paralog (Ward et al., 2010). Consequently, RAD-51 foci may persist on post-synaptic intermediates, reflecting delayed resolution. Although direct evidence in meiotic structures is limited, the conserved dependence of HR protein transport on microtubule dynamics suggests that paclitaxel could interfere with meiotic spindle–or synaptonemal complex–associated repair processes.

In our *C. elegans* experiments, *parp-1, parp-2, and parg-1* transcripts showed no significant changes upon paclitaxel exposure (Figure 4A). Previous studies in mammalian cells similarly reported that paclitaxel alters PARP-1 protein activity, whereas PARP-2 protein levels remain unchanged (Ge et al., 2024). To our knowledge, the transcriptional responses of PARP or PARG to paclitaxel have not been well characterized in either nematodes or mammalian systems. Taken together, our findings suggest that paclitaxel may primarily modulate PARP/PARG activity through post-translational mechanisms, though direct mechanistic evidence remains to be established. Future work examining *parg-1/parg-2* double mutants to investigate potential synergistic effects would help further clarify the respective contributions of PARP and PARG activity to paclitaxel sensitivity.

Vincristine, a microtubule-destabilizing chemotherapeutic, has been reported to induce apoptosis in *C. elegans* via LET-99/GPA-11-mediated activation of JNK-1 (Sendoel et al., 2014). Although our RNA-seq data showed no significant changes in *let-99* or *gpa-11* expression and a modest downregulation of *jnk-1* (log_2_FC = −1.14) upon paclitaxel treatment, we cannot exclude the possibility that paclitaxel affects this pathway at the protein or post-translational level. Thus, apoptosis may still involve protein-level regulation of the LET-99/GPA-11/JNK-1 axis, but our data suggest that transcriptional activation of this pathway is unlikely to be a major contributor in the *C. elegans* germline under these conditions.

### Reduced MMR expression is a stress-induced signature, not a driver, of embryonic lethality

The observed transcriptional downregulation of MMR genes (*msh-2*, *msh-6*, *pms-2*) by RNA-seq and qPCR is intriguing and has also been reported in human cells. However in our *C. elegans* system, neither *msh-2* overexpression, RNAi knockdown, nor *msh-6* mutation altered embryonic susceptibility to paclitaxel. These findings suggest that, in *C. elegans*, MMR downregulation is unlikely to be a primary driver of paclitaxel-induced embryonic lethality. Rather, it appears to represent a stress-associated transcriptional response, possibly linked to apoptosis or global reprogramming. We also note that some of the observed differences could be influenced by general developmental abnormalities or secondary stress effects in treated worms, rather than a direct role of MMR in paclitaxel response. Importantly, this conclusion is specific to the *C. elegans* germline model and does not exclude the possibility that altered MMR activity contributes to paclitaxel resistance or sensitivity in mammalian systems, as reported in several cancer studies. For example, studies in cancer cell lines have reported MSH2 overexpression–associated resistance (Zhang, Yin et al., 2014), indicating that MMR activity may influence drug response differently depending on cellular context.

However, this apparent discrepancy likely reflects context-dependent roles of MMR: in proliferating tumor cells, altered MMR may influence survival pathways and drug responsiveness, whereas in the *C. elegans* germline, paclitaxel-induced embryonic lethality proceeds through mechanisms independent of MMR. This interpretation is consistent with prior studies showing that MMR deficiency does not consistently confer paclitaxel resistance (Fink et al., 1998; Lin and Howell, 1999). Importantly, the reproducibility of such MMR expression changes in *C. elegans* underscores the translational utility of this model for dissecting paclitaxel mechanisms across biological contexts.

Moreover, while we did not directly assess whether deficiencies in other DNA repair pathways or mitotic spindle checkpoint components lead to hypersensitization, our RNA-seq and qPCR analyses showed no significant transcriptional changes in NER, Fanconi anemia, or PARP-related genes. While these pathways might still influence drug responses, investigating their functional roles would be an important direction for future studies.

### Transcriptional reprogramming underlies partial recovery upon multigenerational exposure

RNA-seq analysis revealed upregulation of xenobiotic metabolism genes (e.g., cytochrome P450s) and Toll-like receptor–like signaling, representing canonical stress responses to external insults. Such transcriptional reprogramming may mitigate drug toxicity and contribute to long-term adaptation. Consistent with this, multigenerational assays showed progressive sensitization from F1 to F5, followed by partial recovery in F8/F10. This pattern may reflect either selection of resistant individuals or adaptive transcriptional responses, and our RNA-seq data support the latter by showing activation of detoxification and immune pathways. Nonetheless, further experiments such as lineage tracing or epigenetic profiling will be required to distinguish between these possibilities. Taken together, these findings parallel certain aspects of drug resistance observed in cancer therapy and highlight *C. elegans* as a tractable system for studying long-term paclitaxel adaptation.

### Strengths and future perspectives

This work provides an integrated analysis across multiple biological layers, incorporating morphological (SYP-1 loss, abnormal bivalents), molecular (RAD-51 accumulation, *egl-1* induction), genetic (MMR manipulation), and transcriptomic (xenobiotic/immune pathway activation) evidence. By linking meiosis-specific defects to apoptosis and transcriptional reprogramming, the study establishes a mechanistic framework that extends beyond paclitaxel’s canonical mitotic effects. The multigenerational assays, revealing an initial sensitization followed by partial recovery, further emphasize *C. elegans* as a valuable preclinical platform for studying long-term drug responses and resistance. These findings may inform the understanding of reproductive side effects in chemotherapy and mechanisms of acquired drug resistance.

While broader transcriptional changes, including activation of detoxification and immune-related pathways, were observed, the present study did not aim to comprehensively characterize global transcriptomic remodeling. This represents a limitation of the current work, as additional regulatory pathways beyond mismatch repair may contribute to multigenerational paclitaxel responses. Addressing these broader transcriptional programs will require dedicated transcriptomic analyses and functional validation in future studies. In particular, cell-type–specific and temporal transcriptomic approaches, combined with real-time imaging, will be essential to elucidate non-MMR transcriptional responses to paclitaxel. Additionally, incorporating γH2AX staining and micronucleus assays in mammalian systems will enable a more direct assessment of DNA damage and chromosomal instability associated with paclitaxel-induced transcriptional alterations.

## Data Availability

The original contributions presented in this study are publicly available. These data can be found at: https://www.ncbi.nlm.nih.gov/search/all/?term=PRJNA1423752, accession number PRJNA1423752.
