# Supplementary material for: Paclitaxel-induced germline DNA-damage signatures independent of mismatch repair in C. elegans
Source: Front Pharmacol. 2026 Feb 18;17:1717152. doi: 10.3389/fphar.2026.1717152 (PMC12957893; doi:10.3389/fphar.2026.1717152)
Supplement: Supplementary file 1 [file DataSheet1.docx]

**Supplementary Figures**


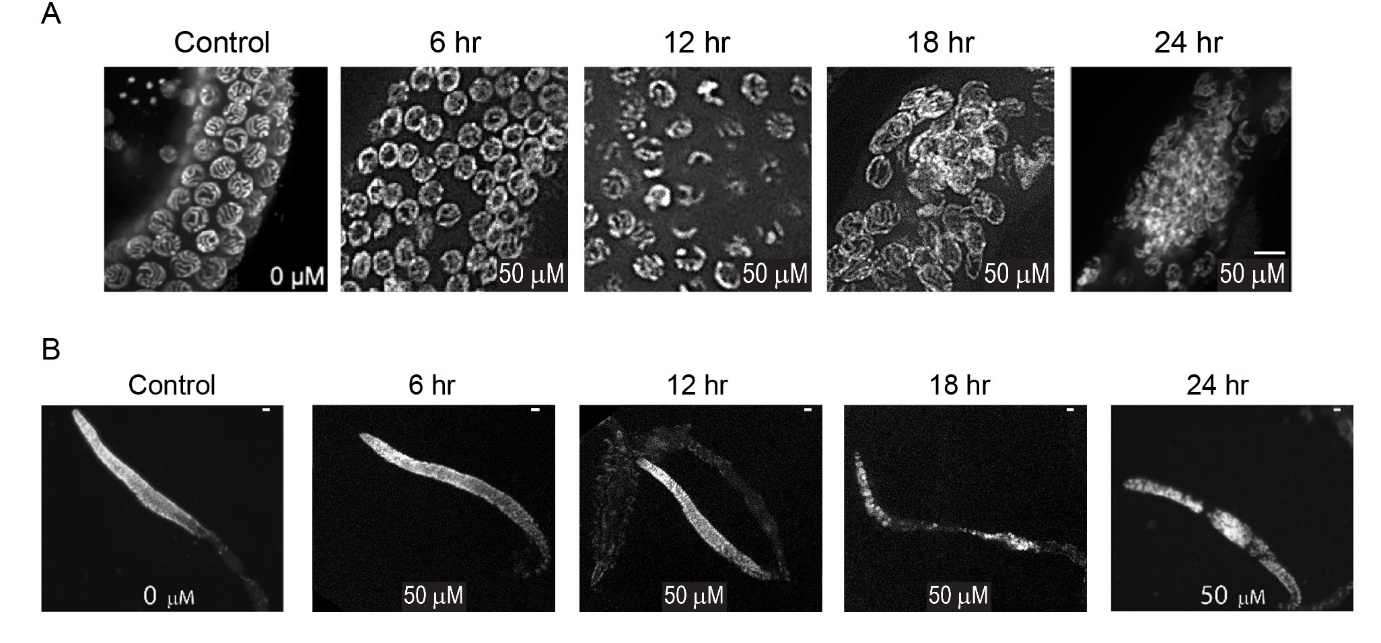


**Supplementary Figure 1. Paclitaxel-induced meiotic defects in *C. elegans* germlines over time.** Untreated control worms displayed well-organized, uniformly spaced nuclei throughout the pachytene region. In contrast, paclitaxel-treated worms exhibited characteristic abnormalities in the pachytene nuclei (A) and in overall gonad morphology (B). See Figure 2 for details. Time-course analysis of worms exposed to 50 µM paclitaxel for 0, 6, 12, 18, and ~24 hours revealed that mild nuclear defects were detectable as early as 6 hours, with pronounced phenotypes observed after 12 hours. N ≈ 15 worms; Scale bars = 50 µm.


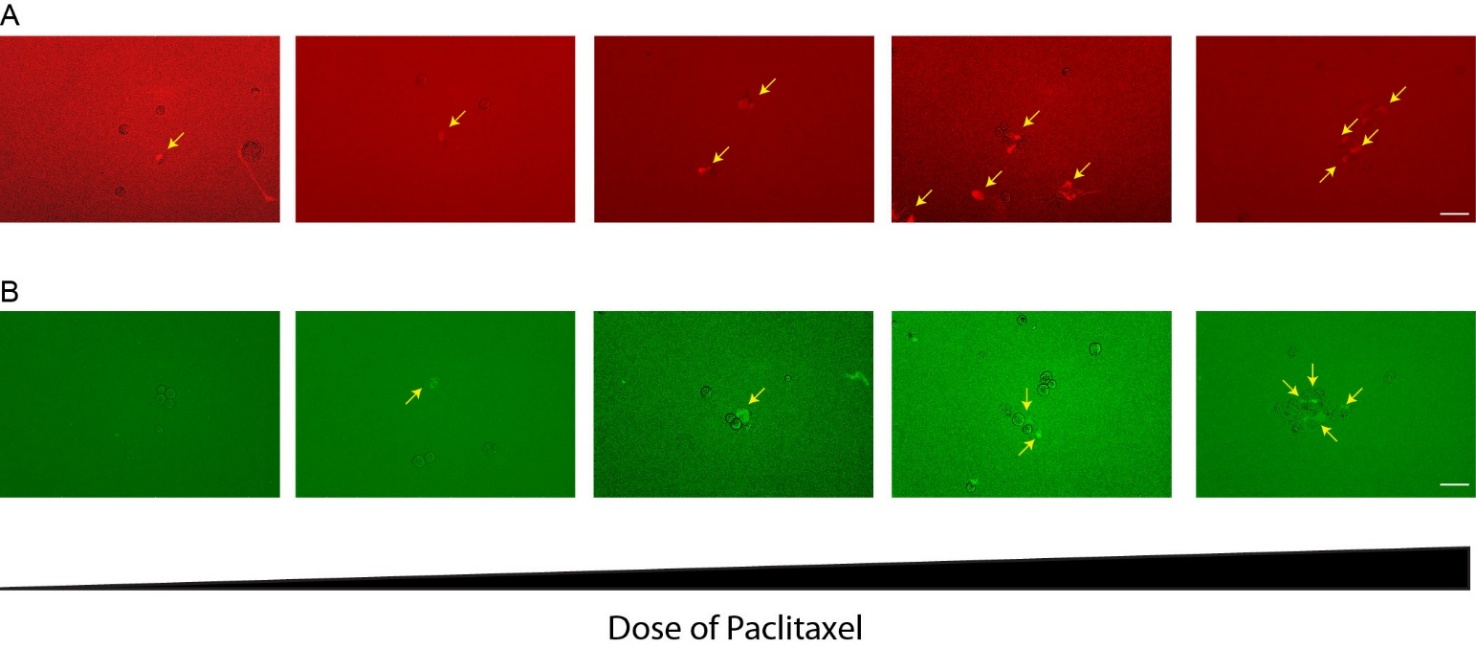


**Supplementary Figure 2. Representative images of paclitaxel-induced cytotoxicity and apoptosis in A498 human renal carcinoma cells.** (A) Dose-dependent cytotoxicity of paclitaxel. A498 cells were treated with increasing concentrations of paclitaxel (0, 5, 15, 25, 50 µM, ~20 h), and membrane-compromised populations were quantified by 7-AAD exclusion. A marked increase in non-viable cells was detected at concentrations ≥ 25 µM. Arrows indicate non-viable cells. Representative images (scale bars = 20 µm). (B) Induction of apoptosis. Annexin V–FITC staining revealed a significant increase in apoptotic fractions in A498 cells treated with 15–50 µM paclitaxel. Arrows indicates apoptotic cells. Data represent images from 3 independent experiments (scale bars = 20 µm).
